# Purchasing Agency

**Published:** 1877-12-20

**Authors:** R. C. Word

**Affiliations:** Managing Editor


PURCHASING AGENCY.—Parties desiring to purchase drugs, or other articles, in Atlanta, may order them through our office at favorable rates. Subscribers to THE RECORD will be charged no commission for this service.

R. C. WORD,
Managing Editor.

---

THE two valuable books reviewed in this issue, to-wit: "Index of Diseases," and "Scudder's Practice," and a variety of medical works, may be found at the store of Messrs. Lynch & Thornton, enterprising book merchants in this city.

